# GPCR Partners as Cancer Driver Genes: Association with PH-Signal Proteins in a Distinctive Signaling Network

**DOI:** 10.3390/ijms22168985

**Published:** 2021-08-20

**Authors:** Jeetendra Kumar Nag, Hodaya Malka, Priyanga Appasamy, Shoshana Sedley, Rachel Bar-Shavit

**Affiliations:** Sharett Institute of Oncology, Hadassah-Hebrew University Medical Center, POB 12000, Jerusalem 91120, Israel; jeetendr.nag@mail.huji.ac.il (J.K.N.); hodaya.malka@mail.huji.ac.il (H.M.); a.priyanga18@gmail.com (P.A.); shanisedley@gmail.com (S.S.)

**Keywords:** G-protein coupled receptors (GPCRs), protease-activated receptors (PARs), angiogenesis

## Abstract

The essential role of G-protein coupled receptors (GPCRs) in tumor growth is recognized, yet a GPCR based drug in cancer is rare. Understanding the molecular path of a tumor driver gene may lead to the design and development of an effective drug. For example, in members of protease-activated receptor (PAR) family (e.g., PAR_1_ and PAR_2_), a novel PH-binding motif is allocated as critical for tumor growth. Animal models have indicated the generation of large tumors in the presence of PAR_1_ or PAR_2_ oncogenes. These tumors showed effective inhibition when the PH-binding motif was either modified or were inhibited by a specific inhibitor targeted to the PH-binding motif. In the second part of the review we discuss several aspects of some cardinal GPCRs in tumor angiogenesis.

## 1. Introduction

Proteases in cancer are long known as tightly associated with tumor progression, facilitating the spread of tumor metastasis. While the world of protease takes a pivotal role in numerous biological processes, unambiguously in cancer biology they are best recognized by their ability to degrade basement membrane barriers, cleavage of structural components of the extracellular matrix as well as proteins that link cancer cells together. As such, the human “degradome”, established by sequencing of the human genome show a great diversity among proteases, generating no less than 569 enzymes which are sub-divided to five subgroups: 194 metalloproteinases, and 176 serine, 150 cysteine, 28 threonine and 21 aspartic proteases [1,2]. Of note are enzymes involved in posttranslational degradation, for instance the deubiquitylases that regulate ubiquitine dependent signaling [3]. Deubiquitylases were recently put on stage, impacting critically on the Wnt/β-catenin signaling path. The attachment of ubiquitine to a cell surface receptor marks it for internalization and degradation through the lysosomal system. For example, the zinc and ring finger 3 (Znrf3) and its homologue ring finger 43 (Rnf43); ZNRF3/RNF43, E3 ubiquitine ligases degrade cell surface frizzled (FZD) receptors and hence regulate the core pathway of Wnt/β-catenin signaling, thereby keeping an adequate and balanced levels of FZDs under normal conditions [4,5]. Deubiquitylases that remove ubiquitine tagging lead to enhanced surface levels of FZDs and induced Wnt signaling in cancer, facilitating tumor growth. However, proteases not only cleave protein substrates, but are capable of sending cellular messages. This takes place by specifically activating cell surface receptors. It refers to serine proteases of the coagulation system that act as powerful “ligands” initiating the activation of protease-activated receptors (PARs), and instigate a selective signaling cascade. Both soluble and matrix-immobilized proteases anchored to the dynamic and flexible tumor microenvironment are engaged to maintain tumor growth and progression [6]. Indeed, a robust crosstalk between proteases and PARs takes place during epithelia tumor progression. PAR family members (PAR_1–4_) belong to the large family of G-protein-coupled receptors (GPCRs). Although the growing appreciation of GPCRs in cancer pathogenesis is emerging, yet untill now, few drugs targeting GPCRs are used in cancer [7,8].

In this review, we will discuss our studies as also research work from other groups on the molecular mechanisms by which members of the coagulation pathway like thrombin, FVIIa and Xa activate GPCR and thereby induce tumor growth and angiogenesis [9,10,11,12]. In the first part we will illustrate how understanding the molecular mechanism of PAR signaling led to the development of PAR based drug discovery.

Large-scale genome-broad evaluations of numerous human tumors have revealed original alterations of GPCRs in cancer. These changes include copy number variations, mutations, methylation alterations and variable levels of gene expression observed in a wide range of cancers [13,14,15]. Yet, the task of establishing a tumor driver gene and therefore an effective drug target is nevertheless a tough challenge ahead. Technological advances in gene-sequencing and bioinformatics led to an explosion of information obtained by tumor biopsy specimen [16,17]. The wealth of information combined with ample gene variations, provide a glimpse to the intricacy of a tumor. Still the issue of how this can be translated to cancer therapeutics is a formidable, difficult task.

A gradual and stepwise approach should be taken. First, it is essential to determine a statistically significant GPCR driver-gene. Next, the selected GPCR must undergo biological testing in an appropriate cancer model system/s. One should keep in mind that genome analysis and the overwhelming flow of information may assist only in the first step, identifying a suitable gene candidate. Elucidation of the molecular pathway and flow of events by an individual or combined gene/s signaling are critical for establishing appropriate building blocks toward a successful intervention and the identification of additional targets for therapy.

The signaling network entails protein modules involved in intermolecular interactions. Among these, the pleckstrin homology (PH) domain is most common. PH domains are known by their structural features combined of an α-helix at the C-terminus of the protein and a seven-stranded β-sandwich [18]. While their folding assembly show a common structural scaffold, PH-domains are of no primary sequence homology. These modules serve as ‘recognition-sites’ acting to connect signal proteins by a specific code or message conveyed through the receptor. Lessons from a survey for the detection of candidates that can assemble with PAR family members, culminated with establishing particular motifs within the tails of PARs that can associate with the PH-domain of signal proteins. These binding motifs proved to be sufficient and critical for PAR based tumor development and therefore assigned as prevailing targets for therapy.

### 1.1. PAR_1&2_ Associate with PH-Signal Proteins via ‘Recognition Motifs’ That Endow Junctional Accesses to Cancer Growth

Both immunoprecipitation and pull-down assays over GST-fused PAR_1_ or PAR_2_ C-tails have indicated the association with Akt/PKB, a serine/threonine protein kinase which plays a central role in tumor cell proliferation, survival and invasiveness [19,20]. This association was mediated via the PH-domain of Akt. Multiple signal proteins carry a PH-domain among of which are; Etk/Bmx, Akt, Vav, SOS1 and GAB1. Akt/PKB was chosen as a representative PH-containing signal protein since Etk/Bmx is not expressed in certain cancer cell lines. The association of Akt with PARs is lipid dependent and generally referred to phosphatidylinositol (3,4,5)-trisphosphate (PtdIns(3,4,5)P3; PIP3) association with PH-signal proteins. Nevertheless, PH-proteins may associate also in a lipid independent manner, directly via protein-protein interactions as was previously demonstrated for Etk/Bmx and Focal Adhesion Kinase (FAK) association [21]. This is also true for PAR_1&2_ association with Etk/Bmx. Hence, PAR motifs can dynamically bind either directly to another protein or dependent on PIP3 lipid moiety, allowing a broader platform of interactions. The critical amino acids of the PAR_2_ PH-binding domain were determined by applying mutants H349A and R352A. It appears that there is a hierarchy for the binding of PAR_2_ C-tail to PH-signal proteins. While PH-Etk/Bmx, PH-Akt and PH-Vav3 bind to the same region of PAR_2_-PH binding motifs, lysates applied on either GST-PH-Etk/BMX, GST-PH-Akt columns have indicated the following outcome. Cancer cell-lines that express endogenously Etk/Bmx preferentially associate with the C-tails of PAR_1&2_. Only in cell lines lacking Etk/BMX the tight association between PAR_1&2_ and Akt is seen [20]. The nature of Etk/Bmx association with PAR_2_ was shown by application of various constructs of *etk/bmx*. When a plasmid in which the PH domain of Etk/Bmx was deleted and replaced by myristoyl group termed Myr-dPH or a construct composed of SH2, SH3 and the kinase domain named; TH-SH2-SH3-KD were evaluated for the binding with PAR_2_, no association was observed with either constructs. In-contrast, a tight association is seen with the *wt* Etk/Bmx. In-addition, application of the PI3K inhibitor; LY294002 did not abrogate association of PAR_2_ with Etk/Bmx, however it markedly attenuated the association of PAR_2_ with Akt. Altogether these results show unequivocally a lipid- independent association of the Etk/Bmx PH-domain with PAR_2_.

### 1.2. Tumor Growth, In Vivo

**Figure 1:**
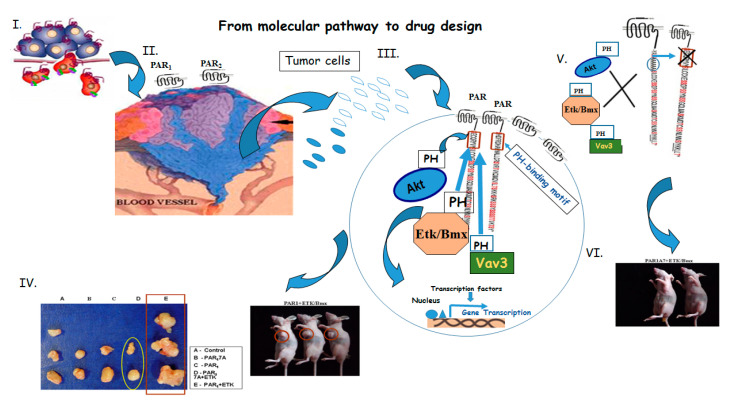
From molecular pathway to drug design. **I**. Scheme of epithelial cells invading basement membrane. **II**. Scheme of tumor cells that invaded the basement membrane and are spreading via the vascular system. **III**. The subfamily of GPCRs; PAR oncogenes display a PH-binding domain within their cytoplasmic C-tails. Signal proteins that possess PH-domain such as; Akt, Etk/Bmx and Vav3 can associate with PAR C-tails initiating thereby a tumor signaling cascade. **IV**. PAR-derived tumors are generated upon the inoculation of cells overexpressing PAR_1_ (or PAR_2_) in the mouse fourth mammary gland. **V**. Application of an inhibitor of the PAR PH-binding domain, effectively abrogates PH-signal protein association with PARs. **VI**. In the presence of the PH-binding site inhibitor, no to nearly very small tumors are developed.

The use of a xenograft mouse model was employed in order to demonstrate the significance of PH-binding motifs in PAR driven tumors. For this purpose, stable clones expressing either *wt Par2*, truncated *Par2* (devoid of the entire cytoplasmic tail), Mut*Par2* H349A or Mut*Par2* R352A clones were generated in HU fibrocystic epithelial cells. When these stable clones were inoculated *s.c.* into nude mice the results obtained showed the importance of PAR_2_ PH-binding motif in tumor growth. While large tumors were seen following inoculation of cells overexpressing *wt Par2*, in-contrast very small tumors were observed in the control HU cell injected as well as following injections of cells exhibiting the truncated form of *Par2* (e.g., incapable of eliciting PAR driven signaling). Inhibition in tumor growth was obtained as well by the implantation of a clone of Mut*Par2* H349A. Tumor measurements of weight and volume as well as by the evaluation of the intensities of the GFP expressed in these tumors, aided to conclude the observed outcome. It is determined that the PH-binding motifs are pivotal for tumor growth in PAR- mediated cancer (Figure 1). PARs are overexpressed in a wide spectrum of epithelial malignancies as opposed to no PAR expression in normal epithelial cells. A partial list of epithelial malignancies include breast-, colon, prostate, lung- and ovarian cancers. We have demonstrated that knocking down of PAR_1_ and/or PAR_2_ expression resulted with significant reduction in breast and prostate tumor growth. It is anticipated that a PAR based therapy will contribute greatly to a wide range of epithelial derived solid tumors. We as also others have demonstrated the overexpression of PAR_1_ for example in prostate cancer [22,23] as also in breast cancer [20]. 

### 1.3. Placenta-EVT Invasion Is Enhanced via PAR_2_-PH Binding Domain

Human pregnancy entails the early-on and well-orchestrated anchorage of the placenta to the uterus decidua. This is mediated by specialized cells called extravillous trophoblasts (EVT). Placenta establishment to the uterus involves the time-limited invasion process which is shut-off thereafter and therefore referred to as a ‘physiological invasion’ process. The importance of PAR_1&2_ PH binding domains in physiological invasion process was demonstrated by an in vitro model based on villi invasion, isolated from early placenta gestation (7–12 weeks of pregnancy). These villi were infected with lentil virus of either *wt hPar1*, mutant *hPar1* 7 A (mutated in the entire PAR_1_ PH-binding domain), *wt hPar2* or *hPar2* H349A and activated with TFLLR (for PAR_1_) or SLIGKV (PAR_2_). Next, the depth of EVT invasion through a Matrigel layer was estimated [15]. While the *wt* constructs showed increased level of invasion with high proliferation rate as indicated by *ki* 67, the mutated PH-binding motif constructs significantly attenuated invasion and proliferation. Hence the PAR_1&2_ PH-binding motifs are essential also for the ‘physiological invasion’ process.

### 1.4. PAR_2_ Is Dominant over PAR_1_

We as well as others [24,25] have shown that PAR_2_ takes a dominant role over PAR_1_ as recapitulated by the essential presence of PAR_2_ for PAR_1_ induced pro-tumor functions. Accordingly, when *sh*RNA silenced *hPar2* was applied, a marked inhibition of PAR_1_ induced function was seen. Consequently, it appears that the signaling by PAR_2_ is pivotal for PAR_1_ function since a truncated form of PAR_2_ devoid of the C-tail, markedly inhibited PAR_1_ induced activities as manifested by colony formation in soft agar, migration and invasion. Co-association between PAR_1_-PAR_2_ is demonstrated by co-IP and confocal analyses indicating the colocalization of PAR_1_ with PAR_2_. This is true also for PAR induced physiological invasion of placenta to the uterus decidua. *sh*RNA*hPar2* significantly inhibited in an organ culture system, the thrombin-activated EVT invasion. Hence, PAR_2_ is also required for PAR_1_ induced EVT invasion. This can be partially explained by the formation of one functional unit entailing PAR_1_-PAR_2_, in PAR-driven invasion, regardless of physiological or pathological invasion process [24,25].

### 1.5. A Lead Cyclic Peptide Directed toward the PAR_2_ PH-Binding Domain

The FDA has approved several cyclic peptide drugs, out of which three; pasireotide, romidepsin and lanreotide are used in cancer. While lanreotide and pasireotide are analogues of the endogenous cyclic peptide hormone somatostatin [26], romidepsin is in-fact a natural product generated from a gram-negative Chromabacterium violaceum and was found useful in T-cell lymphoma [27]. Somatostatin blocks the GPCR somatostatin receptors. These cyclic peptide mimetics have a considerably longer plasma half-life than somatostatin and are utilized to treat endocrine tumors and acromegaly. It is anticipated that a selected cyclic peptide directed toward PAR_2_ PH-binding motif may pave the way to successfully treat solid epithelia malignancies by blocking directly GPCR/PARs as well as possibly inhibiting other signaling network that bypasses and cross-talks with PARs.

Methodology that uses cyclic peptides is valuable and display favorable outcome in treatment. This is ascribed to several favorable properties. First, cyclic peptides possess a large surface region that offers high selectivity and affinity for directed proteins. Second, they constitute little to no toxicity owing to their mild amino acid make-up. Third, cyclic peptides are straightforward to prepare (by automated synthesis), and are pretty easy to handle, alter, and describe, all of which are important properties for therapeutics [28]. Backbone-cyclized peptides and peptidomimetics join to provide an efficient tool combined with pharmacological features and are required for working research implements and therapeutics. Backbone cyclization is a technique that allows the development of cyclic peptides without using amino-acids common to the natural linear peptide, which may be critical for the peptide biological activity, mainly if the peptide is short. The main advantage of this procedure is that the cyclization linkage is formed between backbone atoms, without the side chain active group atoms, which are vital for the peptide link and biological function. This arrangement keeps the regular residue functional groups in their bioactive conformation necessary for drug-like features. A cyclic peptide frequently mimics the protein secondary arrangement and displays sustained and longer stability. Optimization of peptide cyclization usually takes place in terms of ring size and in the field of cancer research for longstanding effects on reducing tumor growth in mice.

### 1.6. Identification of Candidates PH-Domain Binding Motifs in a GPCR Array

**Scheme 1:**
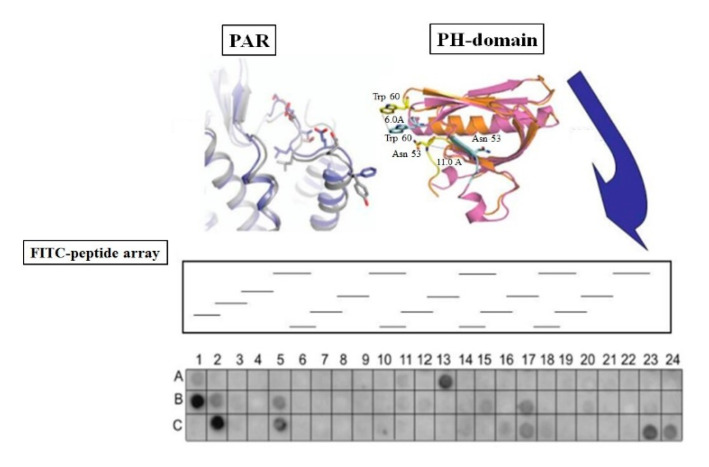
A strategy depicting detection of GPCR PH-binding domain. Application of the purified Akt PH-domain onto an array membrane of cancer driver GPCRs. Scheme of blood vessel. GPCRs expressed in the endothelial cell lining, coagulation factors in the tumor microenvironment. GPCR mediated secretion of angiogenic factors.

In order to allocate powerful cancer therapeutic target sites within candidate GPCRs, it is suggested to isolate the PH-domain of Akt, as a representative of signal protein PH-domains. The PH-domain of Akt and/or Etk/Bmx when sub cloned into a bacteria expression vector pParallel 2-HL T vector between EcoR1 and BamH1 sites enables overexpression of the recombinant DNA inserted. The soluble PH-binding domain can be then tagged with a GFP tag. Next, preparation of an array of GPCR C-tails embedded filter is the best option for filter preparation and a way to minimize loss of secondary structures. Hybridization following application of the GFP tagged-PH-Akt domain alone is intended to allocate PH-binding ‘recognition-sites’ for cancer therapeutic purposes (Scheme 1). This approach will nonetheless assist in finding additional drug targets within GPCRs.

### 1.7. Tumor Angiogenesis

To obtain improved delivery of chemotherapy for the gain of tumor shrinkage, fewer metastases and increased survival time, is yet a tough challenge. Delivery is mediated by the blood vessel network. Keeping in mind that antiangiogenic treatments have not generated extensive clinical benefits, point to the fact that aggressive anti angiogenic strategies and drastic reduction in tumor blood vessels should be re-evaluated. The formation of new blood vessels (vasculogenesis and angiogenesis) includes coordinated functions of endothelial cell migration, proliferation, and tube alignment. Since tumors are limited in size by nutrient diffusion and oxygen supply from neighboring vessels, a wide spectrum of solid tumors are heavily dependent on their vascularization [29,30,31,32]. Certainly, angiogenesis; the growth of blood vessels from pre-existing vessels is the main reason for the flourishing of a tumor, therefore it calls for vessel elimination. On the other hand, drastic reduction of blood vessels impairs proper drug delivery and hence should be carefully monitored. Studies from the lab of Hodivala-Dilke have demonstrated the efficacy of low-dose Cilengitide, a cyclic peptide mimetic of an integrin-specific RGD directed to αvβ3/αvβ5, can markedly improve tumor treatment and stands in-contrast to high-dose Cilengitide. The best outcome was achieved by combination therapy that include low-dose Cilengitide along with Verapamil and the chemotherapy Gemcitabine [33]. This outcome put forward the notion favoring “vascular promotion” for therapeutic purposes. Functional blood vessels must undergo endothelial cell proliferation initiated by Vascular Endothelial Growth Factor (VEGF) and basic Fibroblast Growth Factor (FGF-2; bFGF) which are primary mediators of blood vessels, significantly upregulated in the vicinity of a tumor [31,32]. The appearance of new blood vessels from preexisting one is affected by the growth factor receptors KDR/*flk*-*1* and *flt-1* and by a spectrum of adhesion molecules, primarily integrins [32,34,35].

The pivotal involvement of GPCRs in angiogenesis was demonstrated via Gα13^−/−^ KO mice [36] resulting with mouse embryo lethality. G-proteins are known to play a central part in the cell signaling of GPCRs. Interruption of the gene that encodes Gα13 compromises the ability to generate and build the vascular endothelial system and therefore causes intrauterine embryonal death.

Several GPCRs have been shown as critically impacting on angiogenesis [37,38]. For example, frizzled receptors (FZDs); a GPCR subfamily of the Wnt ligands, are known for governing among others, embryonic stem cell development, pathological malignancies. FZDs 4, 6, 7 and 8 are involved in the development of blood vessels from progenitor embryonic stem cells [39,40,41]. Other GPCRs are best known in participating via the upregulation of VEGF and bFGF. As such are: sphingosine 1P receptor; S1P1) [42,43,44], CXCR4 [45,46,47,48] and PAR_1_ [49].

Exclusive disruption of the S1P1 gene by the Cre/Lox methodology, in the ECs resulted in mouse embryonic lethality. It was demonstrated that the embryonic blood vessels were only partly covered by vascular smooth muscle cells (VSMCs), indicating that S1P1 receptor is critical for vascular maturation [42]. Endothelial differentiation gene; Edg1^–/–^ KO (another name for S1P1) mice showed embryonic hemorrhage and consequently intrauterine death between day E12.5 and E14.5 [50]. S1P1 induces also human vascular (HUVEC) as also bovine aortic vascular endothelial cell (BAVEC) migration, impacting on adult angiogenesis, as well [51].

**Figure 2:**
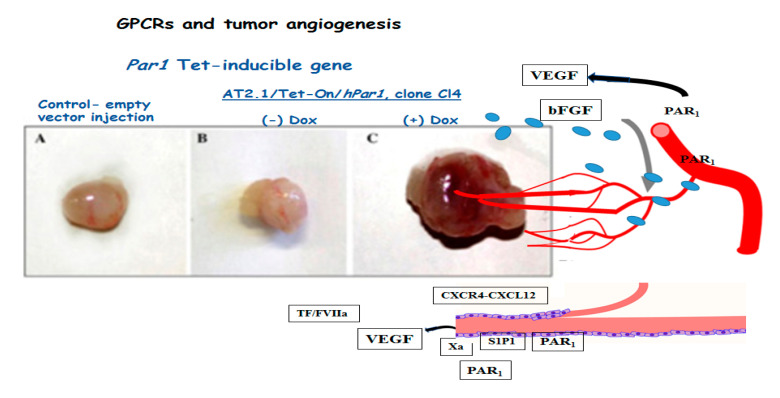
Inducible *Par1* expression in rat prostatic carcinoma increases tumor mass and angiogenesis. AT2.1/Tet-On/*hPar1* clone Cl4 cells and control transfected (**A**) (vector only) cells were injected into rats, subcutaneously. Animals were maintained for 2 weeks with regular drinking water (**B**) or drinking water supplemented with Dox (**A**,**C**). After 2 weeks, tumors were excised and evaluated for weight and color. Tumors shown were from animals injected with the following: (**A**) control transfected cells, nourished with Dox for 2 weeks; (**B**) AT2.1/Tet-On/*hPar1*, clone Cl4; (**C**) AT2.1/Tet-On/*hPar1* clone Cl4, nourished with Dox for 2 weeks.

Studies from our lab have demonstrated that PAR_1_ plays a significant role in the recruitment of blood vessels in vivo using the “Matrigel plug” assay in mice. *Par1* gene recruits effectively blood vessels as shown in animal models. By in vivo injection of Matrigel plugs containing rat prostatic carcinoma cells transfected with the tetracycline-inducible *Par1* an increased angiogenesis and the appearance of reddish Matrigel plugs was observed upon the conditional induced overexpression of *Par1* (Figure 2). It is shown that *Par1* enhances all four splice forms of VEGF, mediated by the signaling events involving PKC, src and PI3K. Functional VEGF was recapitulated by the formation of human endothelial tube network as also bovine aortic endothelial cell proliferation. Oncogene upregulation of Ras, Src and Vav in *Par1* induced VEGF was seen [49]. In this regard, application of a cyclic peptide directed towards the PH-binding domain of PAR may prove beneficial in the combat of PAR induced VEGF mediated angiogenesis.

Along this line of evidence, the group of A. Malik revealed that *Par1* is an essential gene for mouse embryonic stem cell (mESC) differentiation step from progenitor undifferentiated cell status culminating with the formation of blood vessels. This was concluded from an expression profile analysis of GPCRs in mESCs and mESC-derived ECs. In the presence of PAR_1_ differentiation of mESCs to ECs and ultimately the generation of functional blood vessels took place. In-contrast, this differentiation was attenuated upon the depletion of *Par1* [52]. Indeed, studies on PAR_1_^−/−^ KO mice have indicated lethality of ~50% in mouse embryos as a result of impaired vasculogenesis [53]. This outcome is in-line and compatible also with Gα13^−/−^ mouse embryos that are lethal, similar to *Par1* KO embryos [36].

Chemokines and their receptors, principally the chemokine (C-X-C motif) ligand 12 (CXCL12) and its receptor CXCR4, have gained attention for their functional roles as being able to communicate between tumor cells and the tumor microenvironment including immune and vascular cells as also the stroma. They are involved in leukocyte trafficking and numerous pathological conditions. A shared feature in solid tumors and a critical microenvironmental instigator is hypoxia which effectively initiates behavior of aggressive cancers. Under hypoxia, the enhanced overexpression of VEGF [54] is observed as well as the marked upregulation of CXCR4 [55]. This is mediated in a HIF-1α-dependent manner since blocking of HIF-1α attenuated hypoxia induced CXCR4 upregulation. The axis of CXCR4/CXCL12 in angiogenesis is involved in the activation of endothelial progenitor cells as also recruitment of factors such as VEGF [54]. CXCL12 and its receptor enables a permissive microenvironment for angiogenesis, since it has been demonstrated that cancer associated fibroblast inoculated in breast cancer caused enhanced tumor growth and development as compared to normal fibroblasts. This was attributed to the ability of cancer associated fibroblasts to trigger tumor angiogenesis through the recruitment of endothelial progenitor cells facilitated by CXCL12 [56]. The CXCR4 antagonist AMD3100 prevented the ability of CXCL12 to induce high vasculature in glioma by preventing endothelial progenitor cell fusion [57]. Moreover, it is interesting to note that there is a difference between vasculature of breast cancer induced by either *Her2^+^* where anti CXCL12 agents are not effective in contrast to Wnt1 derived tumors. Histological evaluations showed that in MMTV-Wnt1 tumors high levels of epithelial or stromal CXCL12 is observed and therefore anti CXCL12 neutralizing abs effectively inhibited breast cancer derived from MMTV-Wnt1 but not *Her2* induced breast cancer [58].

In conclusion, ample examples of GPCR involvement in tumor angiogenesis and cancer lead to the realization that regulated management of GPCRs and their signaling events may centrally contribute in the combat of cancer.

## Abbreviations

GPCRs	G-protein coupled receptors
PARs	protease-activated receptors
Znrf3	zinc and ring finger 3
Rnf43	ring finger protein 43
FZD	Frizzled
PH	domain-pleckstrin homology domain
FAK	focal adhesion kinase
Gab1-Grb2	associated binder 1
Sos	Son of Sevenless
EVT	extravillous trophoblasts
VEGF	vascular endothelial growth factor
bFGF	basic Fibroblast Growth Factor
mESC	mouse embryonic stem cell
S1P1	Sphingosine-1-phosphate receptor 1
